# Omega-3 polyunsaturated fatty acids alleviate early brain injury after traumatic brain injury by inhibiting neuroinflammation and necroptosis

**DOI:** 10.1515/tnsci-2022-0277

**Published:** 2023-03-06

**Authors:** Yali Wu, Jing Zhang, Xiaoyan Feng, Wei Jiao

**Affiliations:** Department of Neurosurgery, 904th Hospital of Joint Logistic Support Force of PLA, Wuxi, 214044, China; Department of Neurosurgery, The Fourth People’s Hospital of Taizhou, Taizhou, 225300, China; Department of Nursing, 904th Hospital of Joint Logistic Support Force of PLA, 101 Xing Yuan North Road, Wuxi, 214044, China

**Keywords:** omega-3, TBI, early brain injury, neuroinflammation, necroptosis

## Abstract

Presently, traumatic brain injury (TBI) is a leading contributor to disability and mortality that places a considerable financial burden on countries all over the world. Docosahexaenoic acid and eicosapentaenoic acid are two kinds of omega-3 polyunsaturated fatty acids (ω-3 PUFA), both of which have been shown to have beneficial biologically active anti-inflammatory and antioxidant effects. However, the neuroprotective effect of ω-3 PUFA in TBI has not been proven, and its probable mechanism remains obscure. We suppose that ω-3 PUFA can alleviate early brain injury (EBI) via regulating necroptosis and neuroinflammation after TBI. This research intended to examine the neuroprotective effect of ω-3 and its possible molecular pathways in a C57BL/6 mice model of EBI caused by TBI. Cognitive function was assessed by measuring the neuronal necroptosis, neuroinflammatory cytokine levels, brain water content, and neurological score. The findings demonstrate that administration of ω-3 remarkably elevated neurological scores, alleviated cerebral edema, and reduced inflammatory cytokine levels of NF-κB, interleukin-1β (IL-1β), IL-6, and TNF-α, illustrating that ω-3 PUFA attenuated neuroinflammation, necroptosis, and neuronal cell death following TBI. The PPARγ/NF-κB signaling pathway is partially responsible for the neuroprotective activity of ω-3. Collectively, our findings illustrate that ω-3 can alleviate EBI after TBI against neuroinflammation and necroptosis.

## 1 Introduction

Traumatic brain injury (TBI) is a condition that continues to be a serious threat to public health, as well as a primary contributor to disability and mortality that places a huge financial burden on countries all over the globe. The prevalence of TBI is particularly high in low- and middle-income countries [1–3]. Due to a rise in both road accidents (motor vehicle accidents) and the number of older people who experience head injuries through trips and falls, the number of people who suffer TBIs is on the rise [3]. The number of randomized controlled trials has grown over the last several years. Despite great progress in long-term outcomes, medication therapies have failed to demonstrate clear advantages [2–8]. Therefore, it is crucial to investigate novel and efficacious pharmaceutical therapies and get a better comprehension of the pathophysiological mechanisms underpinning TBI. Multiple physiological changes, including but not limited to primary and secondary brain injuries, contribute to TBI-related neuronal death, neurologic deficits, and post-TBI mortality [9]. There are three types of primary brain injuries: disorganization of brain tissue, intracranial bleeding, and damage to the blood–brain barrier, all of which occur directly in the brain tissue, and attempts at prevention are usually futile. On the other hand, secondary brain injuries may be prevented, and this includes lessened calcium influx, oxidative stress, neuronal inflammation, apoptosis, and necroptosis [10,11]. Previous research [12–14] illustrated that reducing cerebral edema and restoring neurological activity following TBI in mice is possible via the suppression of neuroinflammation and necroptosis.

Necroptosis and neuroinflammation in central nervous system (CNS) diseases have a complicated but poorly understood interaction [15,16]. Tumor necrosis factor-alpha (TNFα) and/or Fas ligand stimulation trigger necroptosis, which is distinct from caspase-dependent apoptosis [17]. After activation and interaction of receptor-interacting proteins 1 (RIPK1) and RIPK3, a necrosome complex is formed, after which the mixed lineage kinase domain-like pseudokinase (MLKL) is activated, thus capturing the membranes to produce plenty of inflammatory factors [17–19]. Recent research findings also showed that inhibiting the necroptosis pathway can prevent the process of neuroinflammation [20,21]. However, the link between neuroinflammation and necroptosis in TBI has not been extensively studied. The peroxisome proliferator-activated receptor gamma (PPAR-γ) is a nuclear hormone receptor superfamily member and transcriptional regulator implicated in the modulation of inflammation, differentiation, proliferation, and death [22–24]. These findings suggest that necroptosis after TBI could be linked to the activation of PPARγ/NF-κB signaling and the alleviation of neuroinflammation.

Omega-3 polyunsaturated fatty acids (ω-3 PUFA), which include docosahexaenoic acid (DHA) and eicosapentaenoic acid, are recognized as biologically active compounds that exert anti-inflammatory and antioxidative effects linked to the onset of numerous CNS diseases, like ischemic stroke [25], Parkinson’s disease [26], cardiac arrest [27], subarachnoid hemorrhage [28], TBI [29–31], rodent head injury [32,33], and spinal cord injury [34]. Chen et al. [35] demonstrated that the neuroprotective benefits of ω-3 supplements were observed via suppressing TBI-elicited microglial stimulation and associated inflammation through the regulation of HMGB1 nuclear synthesis and translocation and HMGB1-mediated stimulation of the TLR4/NF-κB signaling pathway. According to a recent study, ω-3 PUFA prevents neuronal death following TBI via the mechanism of upregulating SIRT1-mediated deacetylation of Beclin-1 [36]. Huang et al. [23] indicated that hypoglycemia-induced neuronal necroptosis can be inhibited by DHA, via PPARγ/NF-κB pathway. Montero et al. [37] also confirmed that DHA performs a fundamental neuroprotective role by regulating various cell death mechanisms, including necroptosis, apoptosis, and autophagy. Additional research is warranted to better elucidate the processes via which ω-3 PUFA regulates the necroptosis pathway in TBI. New approaches to treating TBI can be identified by understanding the interplay between neuroinflammation and necroptosis [17,38]. We suppose that ω-3 PUFA can alleviate early brain injury (EBI) by regulating necroptosis and neuroinflammation via PPARγ/NF-κB signaling pathway after TBI.

Herein, we designed a mouse TBI model to examine the influence of ω-3 PUFA on EBI and the interplay between neuronal inflammation and necroptosis. In addition, we examined the mechanism via which the PPARγ/NF-κB signaling pathway regulates this process.

## 2 Materials and methods

### 2.1 Animals

Male C57BL/6J adult mice (22–25 g) (Anhui Medical University, Hefei, China) were used for all investigations. Mice were provided an unrestricted supply of food and water and were preserved in a 12 h light/dark environment.

### 2.2 Experimental design

#### 2.2.1 Experimental design 1

Forty-five mice were randomized into three groups: the sham, TBI, and TBI + ω-3 (*n* = 15/group). Mice in the sham and TBI groups were on a regular diet and with intraperitoneal injection of normal saline, mice in the TBI + ω-3 groups were on the same diet and with intraperitoneal injection of ω-3 PUFA (2 mL/kg, diluted in dimethyl sulfoxide; Sigma, St Louis, MO, USA). ω-3 PUFA was administered once daily for 3 consecutive days, beginning about 30 min after the TBI.

#### 2.2.2 Experimental design 2

Thirty mice were randomized into four groups: TBI + ω-3 and TBI + ω-3 + GW9662 groups (*n* = 15/group). TBI + ω-3 and TBI + ω-3 + GW9662 groups received same dosage ω-3 PUFA. Mice in the TBI + ω-3 group received intraperitoneal injection of normal saline, TBI + ω-3 + GW9662 group received intraperitoneal injection of GW9662 (1 mg/kg, M2748, AbMole) once daily for 5 consecutive days, beginning about 2 days before the TBI.

### 2.3 Animal TBI model

The TBI model was developed in meticulous compliance with the weight-drop model of focal injury developed by Feeney [39,40]. In brief, after intraperitoneally administering sodium pentobarbital (40 mg/kg) to make the mice under anesthesia, they were placed in a brain stereotaxic device. Throughout the procedure, a heating pad was used to maintain the rectal temperature at 37 ± 0.5°C. Subsequently, we made a burr hole in the left hemisphere at the following coordinates: 2.2 mm below the bregma’s horizontal plane, 1 mm lateral, and 0.2 mm posterior. To reveal the dura mater, the bone flap had to be excised. Following this, the dura was positioned underneath a weight-drop apparatus that had an impact sensor. A metal (weight 240 g, tip diameter 3 mm) was dropped from 1 cm above the dura onto the dura mater through a catheter. Then, the scalp was closed, after which the mice were removed from the device. Next, a covering of medical bone wax was applied over the hole. Moreover, the Sham group mice underwent surgical operations similar to those used in the experimental mice, but in the absence of weight-drop impact.

### 2.4 Drug administration

To the TBI + ω-3 animals, intraperitoneal injection of ω-3 PUFA (2 mL/kg, diluted in dimethyl sulfoxide; Sigma, St. Louis, MO, USA) was administered once daily for 3 consecutive days, beginning about 30 min after the TBI [41].

### 2.5 Evaluation of neurobehavioral function

The neurological functioning at 72 h post-TBI was evaluated using a predefined neurological grading system to assess brain damage severity [42,43]. The scoring technique included assessments of motor skills, sensory abilities, reflexes, and balance. Scores were summed to get a total neurological score between 0 and 18. Every mouse was tested for its behavioral characteristics, with higher scores indicating poorer neurological functioning. One observer, who was unaware of the experimental conditions, recorded all the scores for mice behavior (Table S1).

### 2.6 Brain tissue preparation

Mice were anesthetized and underwent perfusion with 200 mL 0.9% normal saline via the heart before being sacrificed by the intraperitoneal injection of sodium pentobarbital (2%; 150 mg/kg). Brain was obtained and placed on ice. A portion of the specimens from experiment 1 and experiment 2 underwent snap freezing and storage at −80°C for ELISA, WB, and RT‑qPCR; whereas, the remaining samples were fixed with 4% formalin overnight at room temperature, embedded in paraffin and cut into 5 µm sections using a paraffin slicer (SLEE medical GmbH) for TdT‑mediated dUTP‑biotin nick end labeling (TUNEL) staining.

### 2.7 Quantification of brain water content

A standard wet–dry technique of brain water content was applied to assess brain edema severity, as has been described earlier [43–45]. At 72 h post-TBI, we euthanized the mice, and their brains were retrieved. The wet weight was measured, and the brains were dried in an oven at 105°C for 24 h until a constant weight was achieved, at which point the dry weight was measured. The percentage of water content (%) in the brain was computed as follows: (wet weight – dry weight)/wet weight × 100%.

### 2.8 Cytokine levels in the ipsilateral cortical tissue

Mice were anesthetized and were transcranial perfused with 50 mL of cold NS. The temporal cortex was collected for assessment. The concentrations of proinflammatory cytokines were detected. There were five supernatants in each group for ELISA analysis. An ELISA was used as directed by the manufacturer to determine the levels of IL‑1β (cat. no. ab197742; Abcam), IL‑6 (cat. no. ab222503; Abcam), TNF‑α (cat. no. ab208348; Abcam), and NF‑κB (cat. no. ab176663; Abcam). The specific experimental procedure according to the guidelines stipulated by the manufacturer and previous study [45].

### 2.9 TUNEL staining

TUNEL assay was carried out to evaluate cell death in the hippocampus. The procedure was performed according to the manufacturer’s instructions with a TUNEL staining kit (Cat# 1684817; Roche Diagnostics GmbH, Basel, Switzerland). After adding the TUNEL reaction solution (50 μL), the slides in each sample were then exposed to a humidified incubation chamber at a temperature of 37°C for an hour. The slides were then examined with the aid of a fluorescence microscope after nuclear staining with DAPI for 5 min at ambient temperature and in the absence of light. The operation was carried out with the help of a TUNEL staining kit following the guidelines stipulated by the manufacturer. Positive cells were observed by microscopy at ×400 magnification. A control without the TUNEL reaction solution was employed as a negative control. The apoptotic index (%) was calculated as the ratio of the number of TUNEL-positive cells/total number of cells × 100. Four high-power fields were chosen at random to verify the cell count, and an average of the data collected from each field was calculated.

### 2.10 Western blot analysis

Previously identified methods for performing western blotting were used [43]. In brief, collecting, homogenizing, and electrophoretically separating cerebral cortex specimens on 10% sodium dodecyl sulfate–polyacrylamide gel electrophoresis were performed. The bicinchoninic acid (BCA) test performed based on a BCA Protein Assay Kit (Beyotime) was utilized to determine the protein concentrations. Following the completion of the separation process, the protein specimens were loaded onto Immobilon nitrocellulose membranes. The next step involved blocking the membranes for 1 h at ambient temperature with 5% nonfat milk. They were subsequently subjected to overnight incubation at 4°C with the following primary antibodies: MLKL (1:1,000, DF7412; Afnity), rabbit anti-NF-κB (1:1,000, rabbit monoclonal, ab32536; Abcam), PPARγ (1:1,000, rabbit monoclonal, ab272718; Abcam), rabbit anti-RIP3 (1:1,000, rabbit polyclonal; Abcam; cat. no. ab62344), rabbit anti-RIP1 (1:1,000, rabbit polyclonal; Abcam; cat. no. ab106393), and rabbit anti-β-actin (1:1,000, rabbit polyclonal, ab8227; Abcam). Following a thrice rinsing of the membranes using TBST, HRP-conjugated goat anti-rabbit IgG or goat anti-mouse IgG secondary antibodies were added at a 1:5,000 dilution to incubate the membranes at ambient temperature for 1.5 h. Bio-Rad imaging equipment (Bio-Rad, Hercules, CA, USA) was utilized to detect the protein bands, and ImageJ software was employed for the subsequent quantification.

### 2.11 Quantitative real-time PCR (qRT-PCR)

Following the procedures outlined previously in literature [46], we conducted qRT-PCR. TRIzol Reagent (Gibco; Thermo Fisher Scientific, Inc., Waltham, MA, USA) was utilized as recommended by the manufacturer for total RNA extraction from cell culture or hippocampus brain tissue samples. Next, the RevertAid First Strand cDNA Synthesis Kit (K1622; Thermo Fisher Scientific Inc., Rockford, IL) was adopted to reverse-transcribe RNA into complementary DNA (cDNA). qPCR conducted with SYBR Green Master Mix (Toyobo Co., Ltd, Osaka, Japan) assessed total TLR4 and NF-κB mRNA levels in the samples. The internal control for the study was GAPDH. The following describes the parameters for the qPCR thermocycling: 45°C (2 min) and 95°C (10 min), followed by 40 cycles of denaturation at 95°C (15 s), annealing at 60°C (1 min), and extension at 72°C (1 min). Each sample was examined three times to ensure accuracy. Below is a list of the gene targets and the particular primers that were used:

NF-κB (forward, 5′-GCGAGAGAAGCACAGATACCA-3′; reverse, 5′-GGTCAGCCTCATAGTAGCCA-3′)

RIP3 (forward, 5′-CCA GAG AGC CAA GCC AAA GAG-3′; reverse, 5′-AGC CAC GGG GTC AGA AGA TG-3′)

MLKL (forward, 5′-CCC ATT TGA AGG CTG TGA TTC TAA G-3′; reverse, 5′-CAG AAA GAC TCC TAC CGT CCA CA-3′)

PPARγ (forward, 5′-CTTTATGGAGCCCAAGTTTGAG-3′ and reverse, 5′-GCTTCACATTCAGCAAACCTG-3′)

GAPDH (forward, 5′-ATGGGTGTGAACCACGAGA-3′ and reverse, 5′-CAGGGATGATGTTCTGGGCA-3′).

### 2.12 Statistical analysis

Mean and SEM are used to present the data. GraphPad Prism 6 (GraphPad Software, San Diego, CA, USA) and SPSS 14.0 (SPSS, Chicago, IL, USA) software were employed to conduct all the analyses of statistical data. Neurological scores are presented as the median and interquartile range. For comparative analysis involving two groups, the student’s *t*-test was used, whereas two independent variables were compared via one-way analysis of variance (ANOVA) followed by Bonferroni’s *post hoc* test. We utilized the Kruskal–Wallis test followed by Dunn’s *post hoc* test for analyzing data that did conform to a normal distribution and/or data with a non-homogeneous variance. *p* < 0.05 denoted the significance criterion for all statistical analyses.


**Ethical approval:** The research related to animals’ use has been complied with all the relevant national regulations and institutional policies for the care and use of animals. The Wuxi Taihu Hospital’s Ethics Committee approved the animal tests involved in this investigation (YXLL-2022-043), ensuring that they conformed to the National Institutes of Health’s recommendations for the handling of lab animals.

## 3 Results

### 3.1 ω-3 ameliorates neurological deficits and cerebral edema following TBI

**Figure 1:**
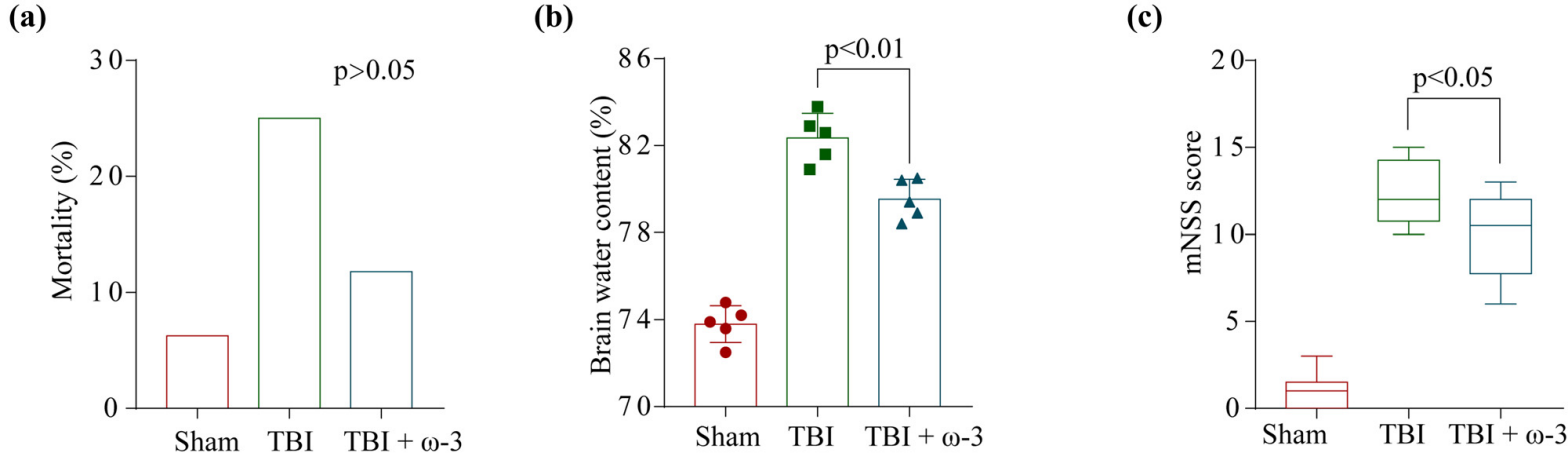
ω-3 alleviates neurological deficits and brain edema after TBI. (a) Comparison of mortality among the three groups (*p* > 0.05). (b) Comparison of the brain water content between the three groups (*n* = 5, *p* < 0.01). (c) Neurological scores of mice in the sham group, TBI group, and TBI + ω-3 group at 72 h after TBI (*n* = 10, *p* < 0.05). ANOVA; mean ± SEM.

To measure brain injury and elucidate the neuroprotection impact of ω-3 on TBI, we computed the modified neurological severity score to assess neurological deficits and assessed the brain water content via the wet–dry technique 72 h following TBI. As depicted in Figure 1, a decrease in the mortality rates (Figure 1a) was noted in the TBI + ω-3 mice, although the difference (variation) was insignificant when compared with the TBI mice (*p* > 0.05). Brain water content rose considerably post-TBI but was reduced after omega-3 therapy (Figure 1b). Correspondingly, neurological scores remarkably dropped post-TBI, and ω-3 treatment remarkably improved the neurological function (Figure 1c).

### 3.2 ω-3 alleviates neuronal damage after TBI

**Figure 2:**
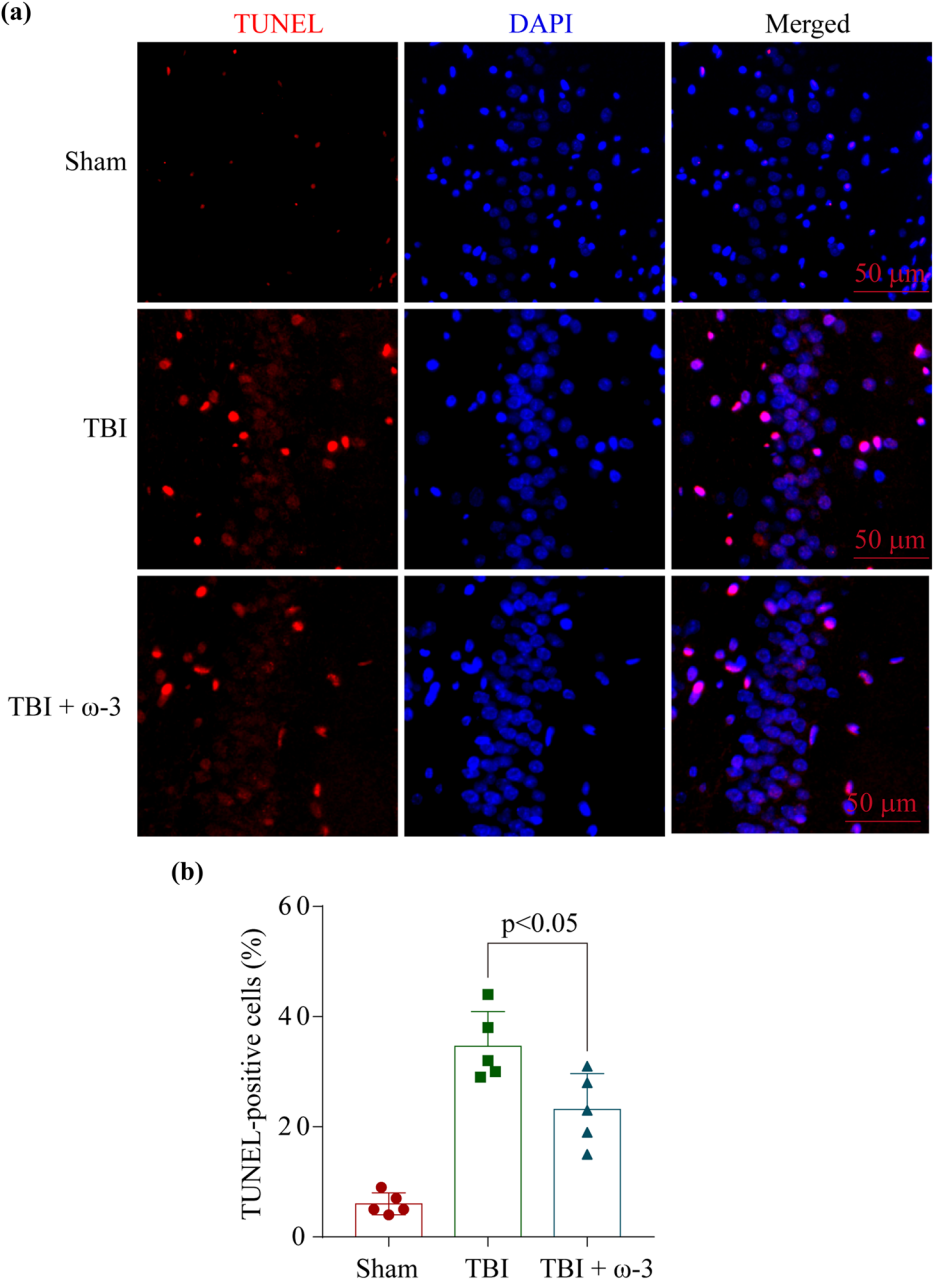
ω-3 alleviates neuronal death after TBI. (a) TUNEL staining showed that ω-3 alleviated neuronal apoptosis in the hippocampus at 72 h after TBI, and (b) representative images of apoptotic neurons are shown. Scale bar = 50 μm, magnification ×200. DAPI, 4′,6-diamidino-2-phenylindole; SAH, subarachnoid hemorrhage; TUNEL, terminal deoxynucleotidyl transferase dUTP nick end labeling.

The primary cause of EBI post-TBI is neuronal death. For this reason, we conducted a TUNEL test to compare the level of cell death in TBI animals following treatment in the absence of the presence of ω-3 at 72 h after the models were established. The data showed that there was an increase in neuronal death in the hippocampus region post-TBI and that the addition of ω-3 attenuated this death and damage (Figure 2).

### 3.3 ω-3 alleviates neuroinflammation after TBI

**Figure 3:**
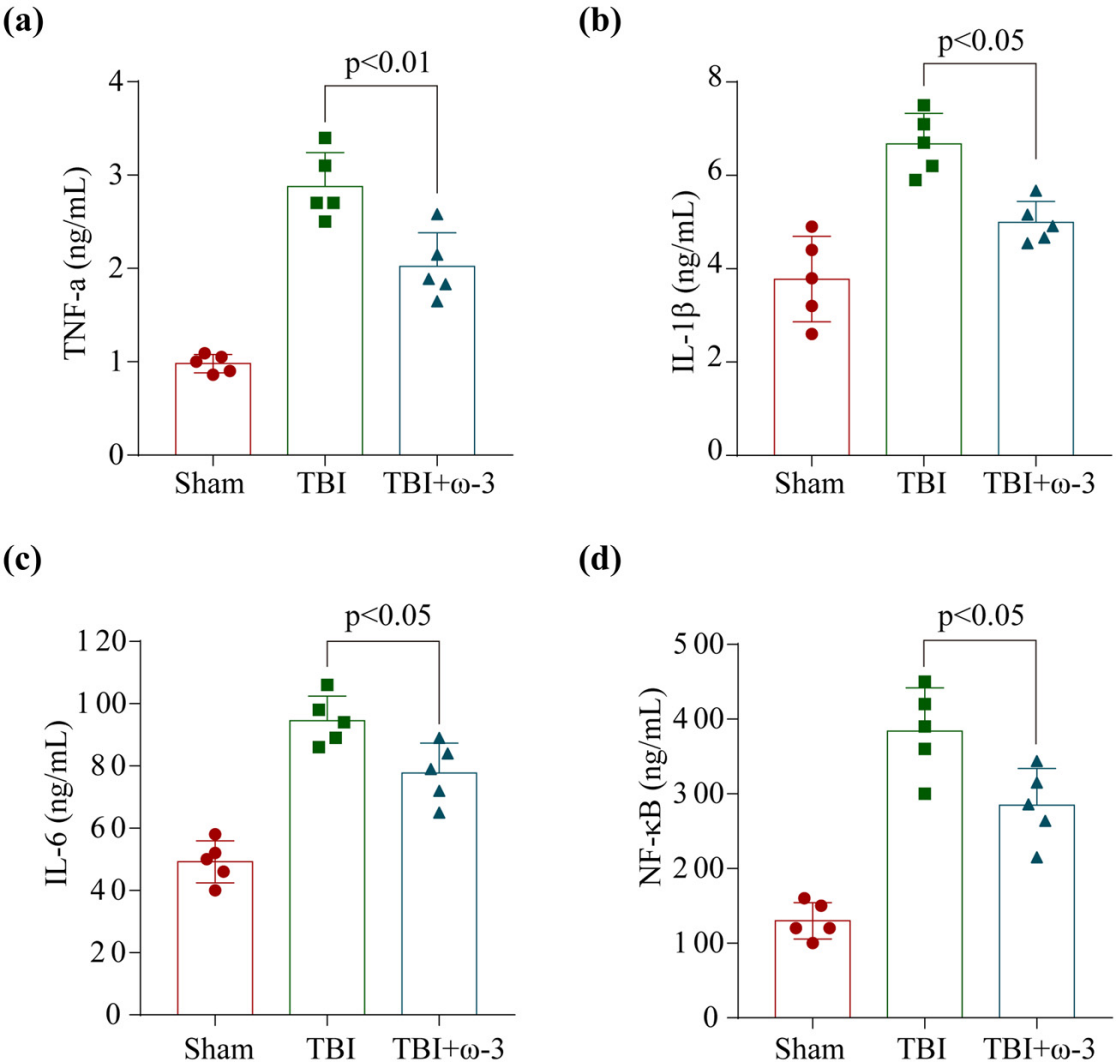
ω-3 alleviates neuroinflammation after TBI. ω-3 significantly reduced hippocampal (a) TNF-α, (b) interleukin-1β (IL-1β), (c) IL-6, and (d) NF-κB levels at 72 h after TBI (*n* = 5, ANOVA; mean ± SEM).

Research has illustrated that neuroinflammation assumes an instrumental function in EBI after TBI and that extensive neuroinflammation might worsen EBI [10,47–49]. Pyroptosis is initiated when pro-inflammatory cytokines such as TNF-α, interleukin-6 (IL-6), and IL-1β are secreted in response to the inflammatory complex and corresponding stimulation of pro-inflammatory signaling through NF-κB. Hence, we employed ELISAs to assess the levels of NF-κB, IL-6, TNF-α, and IL-1β in the hippocampus. Following TBI, the levels of pro-inflammatory cytokines were found to have remarkably elevated, but following treatment with ω-3, their levels were shown to have substantially lowered (Figure 3a–d). Overall, the analysis indicated that ω-3 had powerful anti-inflammatory activity against the neuroinflammation that was generated by TBI.

### 3.4 ω-3 suppresses TBI-triggered necroptosis in the hippocampus

**Figure 4:**
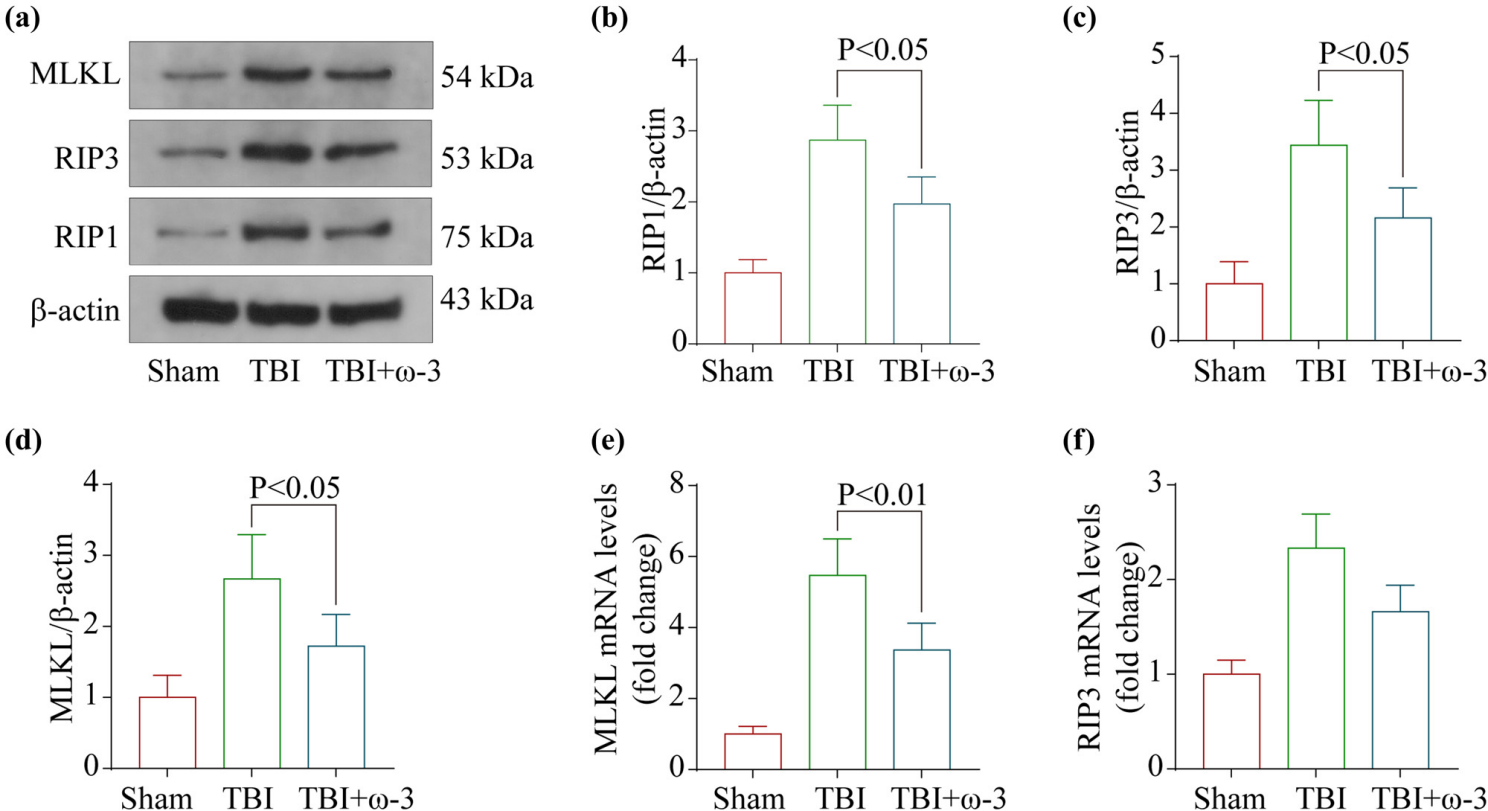
ω-3 inhibits TBI-induced necroptosis in the brain cortex. (a) Levels of MLKL, RIP1, and RIP3 in the brain cortex of mice after TBI were determined using western blotting. (b) Quantification of RIP1 levels in the brain cortex relative to β-actin, the loading control. (c) Quantification of RIP3 levels in the brain cortex relative to β-actin. (d) Quantification of MLKL levels in the brain cortex relative to β-actin. (e) Levels of MLKL mRNA in the brain of TBI mice were measured by real‐time PCR. (f) Levels of RIP3 mRNA in the brain of TBI mice were measured by real‐time PCR (*n* = 5, data are presented as the mean ± SEM, *p* < 0.05).

We determined whether the neuroprotective effects of ω-3 were due to anti-necroptosis mechanisms by detecting the expression profiles of necroptosis-associated protein and mRNA using western blotting and RT-PCR. Subsequently, western blotting was conducted to measure protein expression levels for RIP1, RIP3, and MLKL (Figure 4a). In the TBI group, RIP1, RIP3, and MLKL levels were substantially elevated, but they dropped following ω-3 therapy (Figure 4b–d). In addition, RT-PCR confirmed the same results, supporting the previous findings (Figure 4e–f). In summary, these findings indicated that suppression of the necroptosis signaling pathway could be responsible for the neuroprotective properties of ω-3.

### 3.5 ω-3 modulates neuronal inflammation and necroptosis by regulating the PPARγ/NF-κB signaling pathway post-TBI

**Figure 5:**
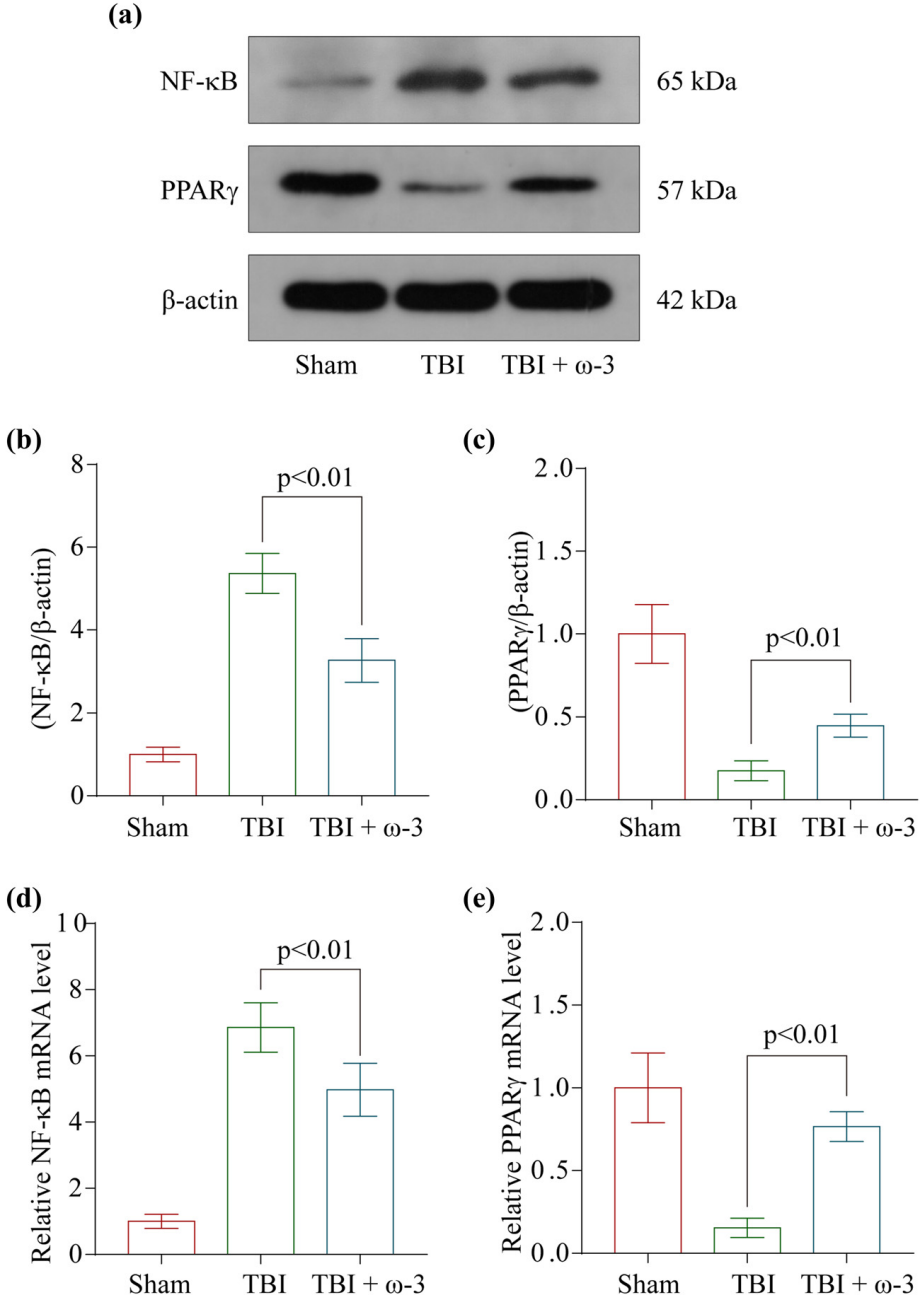
ω-3 regulates neuroinflammation and necroptosis by modulating the PPARγ/NF-κB signaling pathway after TBI. (a) Levels of PPARγ and NF-κB in the brain cortex of mice after TBI were determined using western blotting. (b) Quantification of NF-κB levels in the brain cortex relative to β-actin, the loading control. (c) Quantification of PPARγ levels in the brain cortex relative to β-actin. (d) Quantification of NF-κB levels in the brain cortex relative to β-actin. (e) Levels of PPARγ mRNA in the brain of TBI mice were measured by real‐time PCR (*n* = 5, data are presented as the mean ± SEM, *p* < 0.01).

One of the core signaling pathways implicated in oxidative stress, apoptosis, and necroptosis was PPARγ/NF-κB. We examined whether ω-3’s neuroprotective properties modulate the PPARγ/NF-κB signaling pathway to regulate neuroinflammation and necroptosis post-TBI. We conducted western blotting for determining the PPARγ/NF-κB protein levels (Figure 5a). The levels of PPARγ and NF-κB/p65 were found to be remarkably elevated in the TBI group, whereas they were shown to be lowered following the treatment of ω-3 (Figure 5b and c). In addition, the results of the RT-PCR experiment were quite similar (Figure 5d and e). Overall, these findings confirmed that the neuroprotective effects of ω-3 may be mediated via the regulation of the PPAR/NF-B signaling pathway.

### 3.6 GW9662 reversed the neuroprotection of ω-3 after TBI

**Figure 6:**
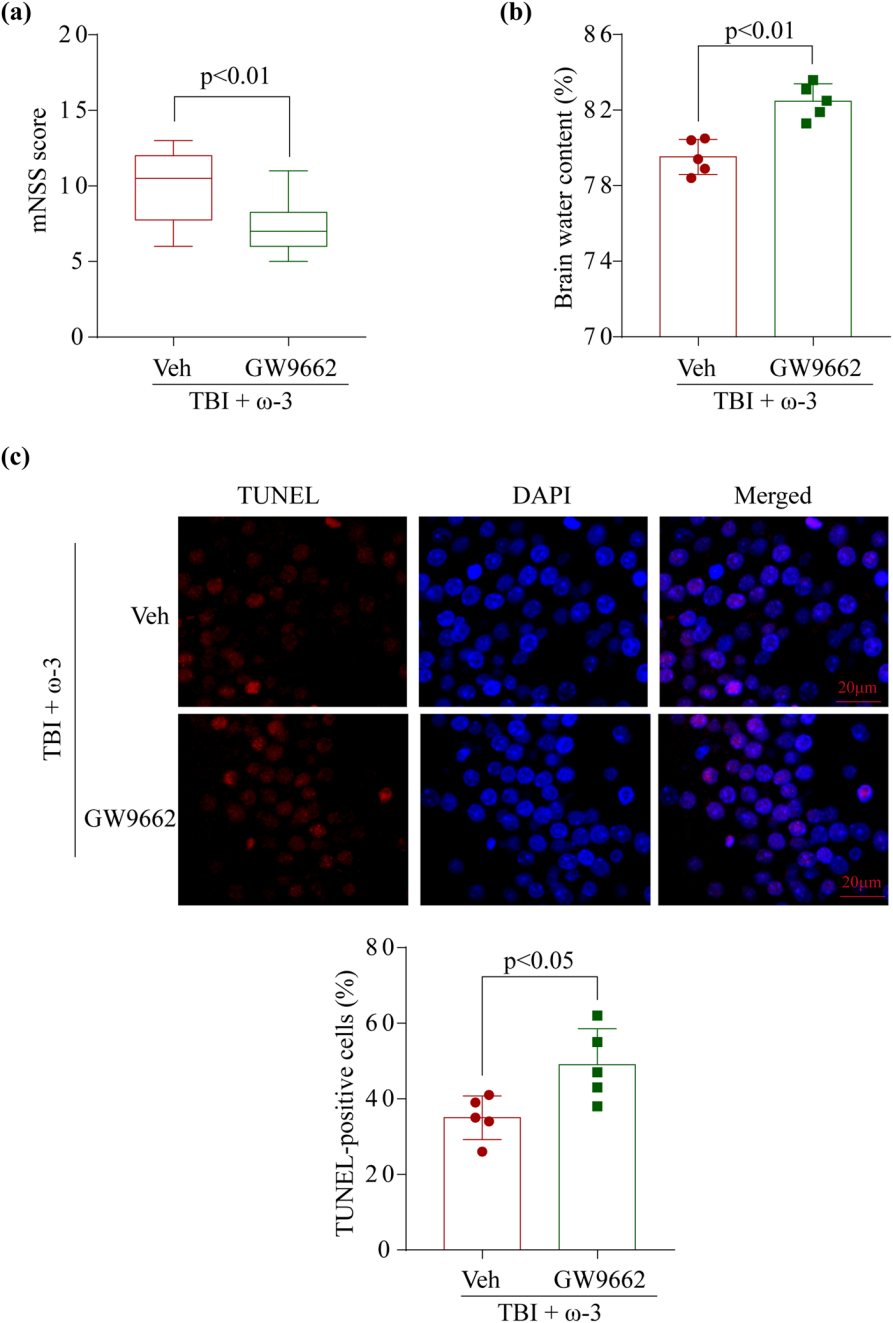
GW9662 reversed the neuroprotection of ω-3 after TBI. (a) Neurological scores of mice in the TBI + ω-3 group and TBI + ω-3 + GW9662 at 72 h after TBI (*p* < 0.01). (b) Comparison of the brain water content between the two groups (*p* < 0.01). (c) TUNEL staining showed that ω-3 alleviated neuronal death in the hippocampus at 72 h after TBI. Scale bar = 20 μm, magnification ×200. DAPI, 4′,6-diamidino-2-phenylindole; SAH, subarachnoid hemorrhage; TUNEL, terminal deoxynucleotidyl transferase dUTP nick end labeling. ANOVA; mean ± SEM.

GW9662 (M2748, AbMole) was used as a PPARγ inhibitor. Before inducing TBI, we pretreated the mice with GW9662 to study the link between the PPARγ/NF-κB pathway and the neuronal protection function of ω-3. The findings demonstrated that pretreatment with GW9662 may considerably worsen neurological deficits (Figure 1c and  6a) and increase the severity of brain edema (Figures 1b and 6b), which consequently caused the neuroprotective action of ω-3 to be rendered ineffective. Furthermore, the TUNEL test demonstrated that GW9662 had the potential to considerably enhance neuronal death in the damaged hippocampal tissues in contrast with the TBI + ω-3 animals (Figures 2 and 6c). Thus, we inferred that GW9662 could inhibit the PPARγ/NF-κB signaling pathway and abrogate the anti-necroptosis properties of ω-3, eventually reversing the neuroprotective effects of ω-3 post-TBI.

## 4 Discussion

Herein, we developed a TBI mouse model to assess the therapeutic significance of ω-3 in ameliorating EBI. The current research demonstrates that ω-3 acts as a neuroprotective factor by reducing EBI post-TBI. We found that ω-3 (1) attenuates neurological deficit caused by TBI, (2) reduces TBI-elicited brain injury in mice, (3) lessens neuroinflammation caused by TBI and, as a result, the degree of damage caused by inflammation in the brain, (4) helps to decrease neuronal death and ameliorates necroptosis post-TBI, and (5) the PPARγ/NF-κB signaling pathway might be linked to the anti-necroptosis properties of ω-3.

ω-3 PUFA is known to protect against ischemic stroke, TBI, intracranial hemorrhage, and models of other neurological disorders [25–28,34]. Recent research has shown that ω-3 may reduce the severity of brain damage caused by ischemia and reperfusion by enhancing neuronal survival, promoting neurovascular regeneration, and stimulating brain remodeling [50]. Cai et al. [51] also reported that DHA administered after a stroke could reduce acute brain damage severity caused by ischemia via the mechanism of skewing the polarization of macrophages toward the M2 subtype. Chen et al. [52] demonstrated that ω-3 PUFA can alleviate brain damage following experimental TBI through the suppression of inflammation by regulating microglia polarity; the associated molecular mechanism was the deacetylation of the HMGB1/NF-κB pathway mediated by SIRT1. Begum et al. [53] confirmed that DHA offers therapeutic applications that might reduce ER stress, aberrant protein accumulation, and neurological deficits if it is given post-TBI. Mills [31,32] and Bailes [30,33] also reported that ω-3 PUFA pretreatment can alleviate EBI after TBI.

The pharmacological effect of ω-3 was more complicated, involving anti-inflammatory properties, immunological modulation, and apoptosis [25–28,34]. The neuroprotective impact of ω-3 post-TBI was numerous and complex. Neuronal apoptosis was inhibited by omega-3 PUFA supplementation, which enhanced autophagy after a TBI [36]. Additionally, ω-3 treatment has been shown to enhance neurological recovery and reduce the severity of white matter damage caused by experimental TBI [54]. Zhu et al. [55] showed in both *in vivo* and *in vitro* that DHA reduces NOX generation in TBI by modulating Nrf2 signaling. The acute single bolus of DHA post-TBI can produce significant improvements in neurological outcome over the long term when given promptly during an acute emergency intervention period; the DHA-derived mediators’ resolvins and protectins are also upregulated, providing additional neuroprotection [56]. It was also noted that DHA administration significantly increased NAD(P)H: quinone oxidoreductase (NQO-1) and heme oxygenase 1 (HO-1) [57]. Our preliminary results also showed that DHA reduces hypoglycemia-induced neuronal damage, which was linked to the activation of the downstream NF-κB pathway, and inhibited the necroptosis pathway [23].

Necroptosis is a regulated mode of cell death that primarily depends on RIPK3 and the MLKL and often manifests with the morphological hallmarks of necrosis [58,59]. Necroptosis does not rely on caspases, unlike apoptosis. The serine/threonine kinases RIPK1, RIPK3, and MLKL have been shown to perform an instrumental function in necroptosis triggered by the TNF [60,61] superfamily, TLR3 or TLR4, and interferon receptors [62]. The mechanisms and molecules modulating neuroinflammation and necroptosis were very complicated and involve NF-κB pathways. In our findings, we confirmed that ω-3 increases the PPARγ levels, subsequently inhibiting the activation of NF-κB and eventually alleviating the activation of neuroinflammation and necroptosis. Through inhibiting necroptosis signaling, DHA reduced TNF-induced cell injury and barrier dysfunction caused by caspase-3 and caspase-8 [63]. In the Alzheimer’s disease model, DHA may block necroptosis of THP-1 cells triggered by Aβ via the RIPK1/RIPK3 signaling pathway [64]. Furthermore, the focus of our work was on mice, and the efficacy of the therapy in people is still up for discussion. Future research will focus on the therapeutic impact of ω-3 on TBI patients.

## 5 Conclusions

In summary, our research demonstrated that PPARγ/NF-κB emerged as a crucial cellular modulatory mechanism and contributed to EBI post-TBI by mediating necroptosis and neuroinflammation. Also, we described that the PPAR/NF-B pathway regulates necroptosis via the action of ω-3 and offered a fresh perspective on the biological effects and mechanisms behind the ω-3’s anti-inflammatory, anti-necroptosis, and neuroprotection properties.

## Supplementary Material

Supplementary material
