# Supplementary material for: Omega-3 polyunsaturated fatty acids alleviate early brain injury after traumatic brain injury by inhibiting neuroinflammation and necroptosis
Source: Transl Neurosci. 2023 Mar 6;14(1):20220277. doi: 10.1515/tnsci-2022-0277 (PMC9990778; doi:10.1515/tnsci-2022-0277)
Supplement: Supplementary material [file tnsci-2022-0277-sm.pdf]

Supplementary material

Table S1: Neurological behavior scores

Category	Behavior	Score
Spontaneous activity	Moved around, explored the environment, and approached at least three walls of the cage	3
	Slightly affected moved, did not approach all sides, move hesitating, moved to least one upper rim of the cage	2
	Severely affected moved,did not rise up at all and barely moved in the cage	1
	Did not move at all	0
Symmetry in the movement of four limbs	All four limbs extended symmetrically	3
	Limbs on left side extended less or more slowly than those on the right	2
	Limbs on left side showed minimal movement	1
	Forelimb on left side did not move at all	0
Forepaw outstretching	Both forelimbs were outstretched, forepaws walked symmetrically	3
	Left side outstretched less than the right, and forepaw walking was impaired	2
	Left forelimb moved minimally	1
	Left forelimb did not move	0
Climbing	Climbed easily and gripped tightly to the wire	3
	Left side impaired while climbing or did not grip as hard as the right side	2
	Failed to climb or tended to circle instead of climbing	1
Body proprioception	Reacted by turning head and was equally startled by the stimulus on both sides	3
	Reacted slowly to stimulus on left side	2
	Did not respond to the stimulus placed on the left side	1
Response to vibrissae touch	Reacted by turning head or was equally startled by the stimulus on both sides	3
	Reacted slowly to stimulus on left side	2
	Did not respond to stimulus on the left side	1
